# Triage policy to postpone endoscopy for patients with low-risk varices is safe during the lockdown period of COVID-19 pandemic

**DOI:** 10.1186/s12876-023-02866-5

**Published:** 2023-07-12

**Authors:** Yu-Jen Chen, Ming-Chih Hou, Tsung-Chieh Yang, Pei-Chang Lee, Yi-Hsiang Huang, Fa-Yauh Lee

**Affiliations:** 1 Division of Gastroenterology and Hepatology, Department of Medicine, Taipei Veterans General Hospital, No. 201, Sec. 2, Shih-Pai Rd., Taipei 112, Taipei City, Taiwan; 2 National Yang-Ming Chiao-Tung University School of Medicine, Taipei, Taiwan, ROC

**Keywords:** COVID-19, Esophageal varices, Triage policy

## Abstract

**Background & aims:**

During the COVID-19 pandemic, most of the endoscopic services were electively postponed or suspended. We aimed to assess the safety of a triage policy in patients receiving esophageal variceal ligation during the COVID-19 pandemic.

**Methods:**

Triage policy of endoscopic variceal ligation (EVL) was implemented in our hospital during the lockdown period from 15th May 2021 to 26th July 2021. One experienced gastroenterologist reviewed the prior-scheduled list of patients for the EVL prophylaxisprogram. We compared the clinical characteristics and outcomes with those receiving endoscopy due to esophageal varices from 17th May 2020 to 28th July 2020.

**Results:**

Of the 124 patients receiving EVL, a higher percentage of esophageal variceal bleeding (EVB) was noted (9/32, 28.1% vs. 8/92, 8.7%, p = 0.006) during the lockdown period, with a higher percentage of EVB in the referrals (7/9, 77.8% vs. 2/14, 14.2%, p = 0.007). Among patients who received prophylactic EVL, 6 of 78 (7.7%) experienced EVB during the normal period, which is no different to 2 of 23 (8.7%) during the lockdown period. Twenty-three patients whose endoscopies were postponed by triage policy due to low-risk or eradicated varices did not experience EVB during the lockdown period. Child-Turcotte-Pugh (CTP) class C was predictive of EVB (relative risk 8.400, P = 0.033), entering the program of prophylactic EVL was the protective factor of EVB (relative risk 0.016, P = 0.002).

**Conclusion:**

Entrance into the prophylaxis program does not only decreases risk of EVB but also fosters comprehensive triage to postpone endoscopy during the lockdown period.

## Introduction

The COVID-19 pandemic, caused by severe acute respiratory syndrome coronavirus-2 (SARS-CoV-2), changed people’s lives around the world. In many countries, once the community epidemic occurred, the government would announce the lockdown strategy, leading to reduction in medical services as well [1, 2].

Considering that the virus spreads primarily through droplets and aerosols [3], they could also be expelled during endoscopy examination [4]. As such, triaging patients undergoing endoscopy was suggested by the European Society of Gastrointestinal Endoscopy (ESGE) and the American Society of Gastrointestinal Endoscopy (ASGE) [5, 6] to minimize risk of COVID-19 infection.

Decreased endoscopy volumes or delayed schedules were observed in many countries during lockdown periods, such as China, The Netherlands, the United Kingdom, France, Italy, and the United States [7–12]. However, reduction of endoscopy volumes might be accompanied by unintended consequences. Training of gastroenterologists was interrupted [12]. Absolute detection of cancer decreased during the lockdown in the Netherlands [8], the United Kingdom [9] and the United States [13], accompanied by an increasing proportion of advanced cancer using endoscopic diagnosis. This implied delayed diagnosis of gastrointestinal tract cancer, due to the mitigation of endoscopic procedures during lockdown. In the same way, the likelihood of detecting acute upper gastrointestinal bleeding during gastroscopy increased during lockdown [14]. Furthermore, patients received endoscopy for upper gastrointestinal bleeding have reduced 30-day survival during lockdown period [15]. These findings suggest that the severity and clinical outcome of gastrointestinal disorders, diagnosed by endoscopy could have been influenced due to the lockdown policy.

Portal hypertension is a common complication of liver cirrhosis. Cirrhotic patients with portal hypertension are in high risk of developing esophageal varices (EVs). In patients with high-risk varices, primary prophylaxis for related bleeding with ligation or non-selective beta blockers (NSBBs) were recommended by the American Association for the Study of Liver Diseases and the European Association for the Study of the Liver [16, 17]. Furthermore, secondary prophylaxis with band ligation along with NSBBs was suggested in patients who had experienced prior variceal bleeding according to latest guideline [18]. Serial band ligation is usually deployed for these patients to eradicate EVs. On the other hand, the US Veteran Health Affairs (VHA) guidance [19] recommended esophageal variceal ligation (EVL) as an elective and non-urgent procedure for resumption of endoscopic services during the COVID-19 pandemic. As we know, the bleeding rate of high-risk varices is 15% annually [20] and the six-week mortality rate is about 20% among patients with variceal bleeding [21]. Considering that non-urgent procedures could be postponed for several months during lockdown, the impact of medical service reduction should be identified in such vulnerable patients.

There were few indigenous cases of COVID-19 and no evidence of community transmission in Taiwan until May 2021. The Central Epidemic Command Center (CECC) in Taiwan announced a level 3 epidemic warning for Taipei City and New Taipei City on May 15 2021, then it was announced nationwide on May 19, 2021 [22, 23]. The level 3 epidemic warning included the closure of leisure, entertainment venues, and educational facilities; family or social gatherings involving five or more people indoors or 10 or more people outdoors were suspended. The CECC also asked medical institutions to reduce routine medical services [24]. A number of scheduled endoscopy procedures were postponed or cancelled. As the community outbreak subsided, the CECC downregulated epidemic warnings to level 2 on July 27, 2021, allowing medical institutions to resume routine services [25].

In this study, we tried to assess the impact of mitigated endoscopies with implementation of a triage policy in cirrhotic patients undergoing a predefined schedule of EVL during the lockdown period. We also compared the clinical characteristics of cirrhotic patients undergoing EVL in the lockdown period and the normal period without reduction of endoscopic services.

## Materials and methods

### Patients

We retrospectively reviewed 1,586 esophagogastroduodenoscopies (EGDs) from May 15, 2021, to July 26, 2021 (10 weeks), which was defined as a lockdown period. Comparatively, 4,902 EGDs were reviewed from May 17, 2020, to July 28, 2020 (10 weeks), defined as a normal period. Although the latest Baveno VII consensus [26] preferred NSBBs over EVL in prevention of first variceal bleeding, the renewing consensus had not been published during our study period. Thus, we followed the recommendation of Baveno VI. According to Baveno VI consensus [27], either NSBBs or endoscopic band ligation is recommended for the prevention of the first variceal bleeding of medium or large varices. In our hospital, the patients would enter EVL prophylaxis program either for primary prophylaxis or secondary prophylaxis, after their first EVL, they would be followed every month to receive an EGD or ligation if required until varices were eradicated. After that, EGD would be performed twice every 3 months, and then every 6 months. If there was no recurrence of esophageal varices, EGD would be followed annually. Urgent EVL would be performed for referred patients due to high-risk varices or acute EVB. All patients were followed until Dec 31, 2022.

During normal period, prior scheduled endoscopy would be arranged on time. In contrast, triage policy was implemented in our hospital during the lockdown, with one experienced gastroenterologist who reviewed the prior-scheduled list of patients for the EVL prophylaxis program before procedure. Only patients with high-risk EVs received EVL, otherwise, the endoscopies for those with low-risk EV or eradicated EV were postponed. The evaluation of EVs was based on previous endoscopy images. All postponed endoscopies were re-scheduled after the lockdown was ended, as announced by the government. Clinical characteristics, including the cause of liver cirrhosis, association with hepatocellular carcinoma (HCC), or other malignancies, and prescription of NSBBs were recorded within 3 months of endoscopies. All laboratory data including complete blood count, renal, hepatic, and coagulation function, and serum level of albumin were recorded.

The presence of EV was assessed by EGD and classified as F1, small and straight varices; F2, moderately sized, tortuous varices; and F3, large, tumorous varices. EV with the size of F2 and F3, or F1 with red coloring, was defined as high-risk EV [28]. Variceal bleeding was defined by active bleeding, and white nipple sign, with upper gastrointestinal tract bleeding and large varices, but no other potential bleeders. The Albumin-Bilirubin (ALBI) score was calculated as: (log_10_ bilirubin [µmol/L] × 0.66) + (albumin [g/L] × −0.0852). ALBI grade 1, 2, and 3 were stratified as follows: ALBI score ≤ − 2.60 (ALBI grade 1), > −2.60 to ≤ − 1.39 (ALBI grade 2), and > − 1.39 (ALBI grade 3) [29]. The Platelet-albumin-bilirubin (PALBI) score was calculated as: (2.02 × log_10_ bilirubin) + [-0.37 × (log_10_ bilirubin)2] + (-0.04 × albumin) + (-3.48 × log_10_ platelets) + [1.01 (log_10_ platelets)2], where bilirubin is measured in µmol/L and albumin in g/L, and platelet count in 1000/µL. PALBI grade was categorized as: PALBI grade 1 (Score ≤ 2.53), PALBI grade 2 (Score > 2.53 and ≤ 2.09), and PALBI grade 3 (Score > 2.09) [30].

### Ethics approval and consent

The study was executed in accordance with the Declaration of Helsinki and was approved by the Institutional Review Board of Taipei Veterans General Hospital (VGHIRB No. 2021-12-005CC). Consent waivers were obtained, and patient’ records were anonymized and de-identified prior to analysis.

### Statistical analysis

The primary endpoint of this study was EVB. The Fisher exact test or a χ^2^-test with a Yates correction was performed for categorical variables, and the Mann–Whitney U-test was performed for continuous variables. The variables with statistical significance (P < 0.05) or approximate significance (P < 0.1) by univariate analysis. Multivariate analysis were not performed due to limited events. A two-tailed value of P less than 0.05 was statistically significant. All statistical analyses were carried out by using IBM SPSS-IBM Statistics for Windows, version 23.0 (IBM Corp., Armonk, NY, USA).

## Results

### The flow of endoscopy management in the normal and lockdown period

**Fig. 1:**
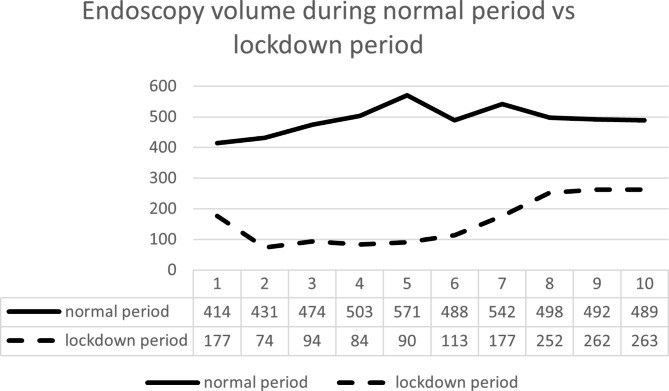
Endoscopy volume during the normal period vs. the lockdown period

**Fig. 2:**
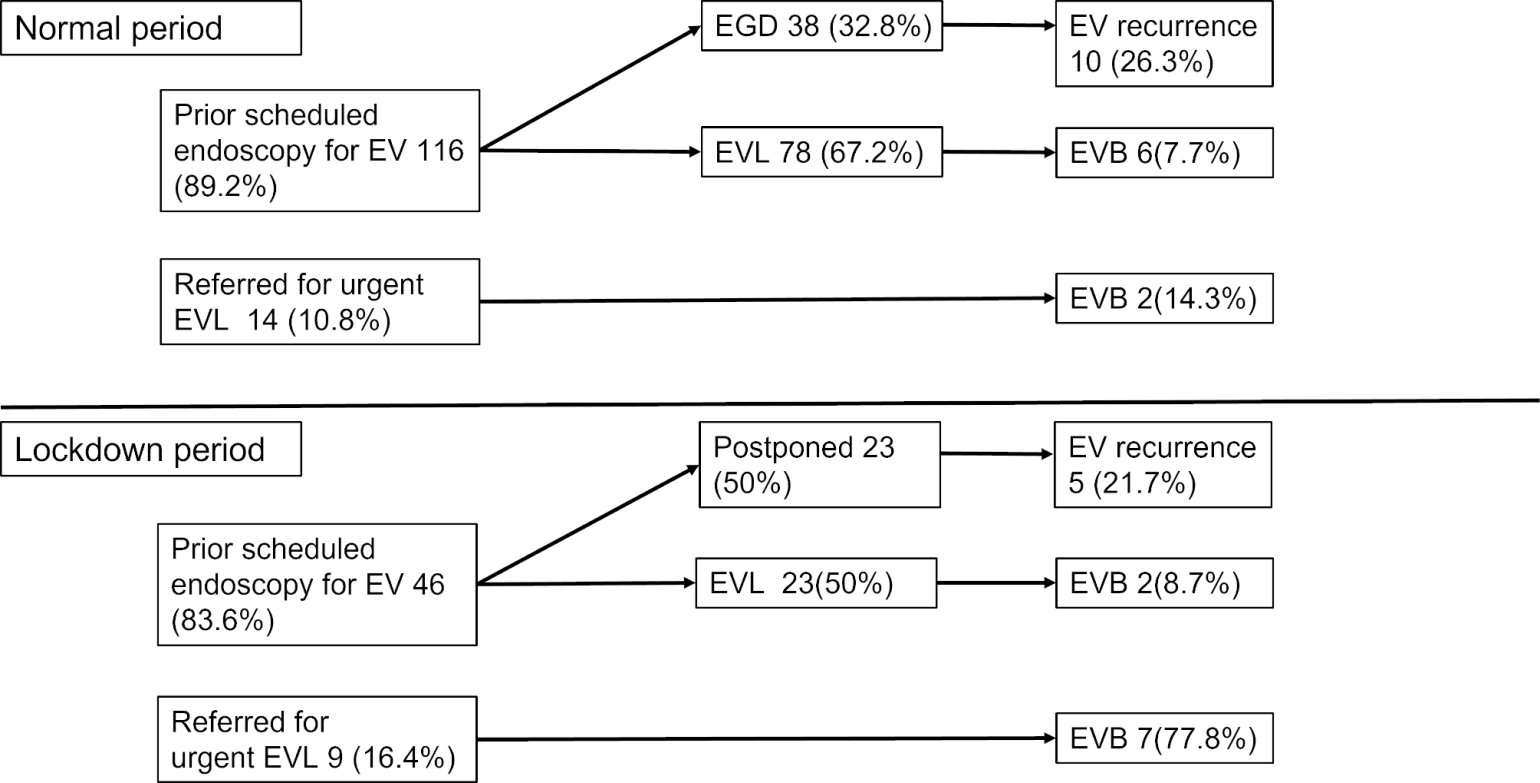
Patients’ flow of endoscopy management in the normal and the lockdown period

There were 4,902 EGDs performed during the normal period in comparison to 1,586 during the lockdown period (Fig. 1). In all, there were 185 patients scheduled to undergo serial EVLs, 124 eventually receiving EVLs in these two periods. In the normal period, endoscopic management of EV was requested for 130 patients, including 116 (89.2%) for prior-scheduled prophylactic EVLs and 14 (10.8%) referred patients for urgent EVL due to high risk varices or EVB. Of 116 patients, 78 (67.2%) underwent EVL, with 38 (32.8%) patients eventually received EGD without ligation due to eradicated EV. Six patients experienced bleeding before scheduled EVL. Among 14 referrals for urgent EVL, 4 of 14 (28.5%) were under NSBB and did not have EVB; 2 of the 10 patients without previous primary prophylaxis of EV experienced EVB. During lockdown, endoscopic management of EV was requested for 55 patients, including 46 (83.6%) for prior-scheduled prophylactic EVL and 9 (16.4%) referred for urgent EVL due to high risk varices or EVB. Of 46 patients, EVL was prioritized for 23 patients (50%) due to high-risk varices, while 23 patients postponed endoscopy due to their small or eradicated varices. Two (8.7%) of 23 patients experienced bleeding before scheduled EVL. Seven (77.8%) of 9 referrals had EVB. Excluding referrals, EV bleeding occurred in 6 of 116 (5.2%) during the normal period vs. 2 of 46 (4.3%) during the lockdown period (Fig. 2). In the postponed group, no patient experienced EVB during 18 months follow-up period. However, 5 of 23 patients (21.7%) postponed endoscopy due to their small or eradicated varices during the lockdown period experienced EV recurrence; while 10 of 38 (26.3%) patients in normal period with small or eradicated varices experienced EV recurrence (Fig. 2).

### Clinical characteristics of patients undergoing EVL

Of 124 patients undergoing EVL, there were 15 (12.1%) with CTP class C hepatic function, 93 (75%) with high-risk varices, 52 (41.9%) with the use of NSBBs, and 101 (81.5%) entering into an EVL prophylaxis program, the other 23 (18.5%) patients were referred for urgent EVL due to high risk varices or EVB. A higher percentage of EVB was noted (9/32, 28.1% vs. 8/92, 8.7%, p = 0.006) during the lockdown period in comparison to the normal period. There were no difference in gender, hepatitis B infection, hepatitis C infection or alcohol use between patients received EVL in the normal period and lockdown period. Although there were more patients with MELD score > 10 (71.9% vs. 54.3%), advanced CTP class (CTP A: 46.9% vs. 63%), higher ALBI grade (ALBI grade 1: 9.1% vs. 28.3%) and higher PALBI grade (PALBI grade 1: 18.7% vs. 35.9%) in lockdown period, there was no statistical significance. However, patients’ serum albumin was lower and the aspartate transaminase (AST) level was higher in lockdown (Table 1). There was also a higher percentage of EVB in the referrals during the lockdown period (7/9, 77.8% vs. 2/14, 14.2%, p = 0.007). Among patients who received prophylactic EVL, 6 of 78 (7.7%) experienced EVB during the normal period, which is no different to and 2 of 23 (8.7%) during the lockdown period.

**Table 1:** Demographic data of patients received EVL in the normal period and the lockdown period

Patient Demographic	All(N = 124)	The normal period(N = 92)	The lockdown period(N = 32)	*p* value
Age(years)	63(16–89)	64(16–89)	60(35–87)	0.436
Sex				0.638
Male	85(68.5%)	62(67.4%)	23(71.9%)	
Female	39(31.5%)	30(32.6%)	9(28.1%)	
HBsAg				0.887
Positive	40(32.2%)	30(32.6%)	10(31.3%)	
Negative	84(67.8%)	62(67.4%)	22(68.7%)	
Anti-HCV				0.275
Positive	21(16.9%)	18(19.6%)	3(10.3%)	
Negative	103(83.1%)	74(80.4%)	29(89.7%)	
Alcohol				0.615
Positive	26(21.0%)	18(19.6%)	8(25%)	
Negative	98(79.0%)	74(80.4%)	24(75%)	
MELD score > 10				0.098
Yes	73(58.9%)	50(54.3%)	23(71.9%)	
No	51(41.1%)	42(45.7%)	9(28.1%)	
CTP class				0.217
A	73(58.9%)	58(63.0%)	15(46.9%)	
B	36(29.0%)	25(27.2%)	11(34.4%)	
C	15(12.1%)	9(9.8%)	6(18.7%)	
ALBI grade				0.094
1	29(23.4%)	26(28.3%)	3(9.4%)	
2	82(66.1%)	57(62.0%)	25(78.1%)	
3	13(10.5%)	9(9.7%)	4(12.5%)	
PALBI grade				0.153
1	39(31.5%)	33(35.9%)	6(18.7%)	
2	41(33.1%)	27(29.3%)	14(43.8%)	
3	44(35.4%)	32(34.8%)	12(37.5%)	
HCC				0.073
Yes	35(28.2%)	30(32.6%)	5(15.6%)	
No	89(71.8%)	62(67.4%)	27(84.4%)	
Other malignancy				0.049
Yes	9(7.3%)	4(4.3%)	5(15.6%)	
No	115(92.7%)	88(95.7%)	27(84.4%)	
Biochemistry				
Albumin (g/dl)	3.5(2.1–4.8)	3.6(2.5–4.8)	3.3(2.1–4.4)	0.035
ALT (IU/L)	29.5(5-254)	28(5–87)	37(15–254)	0.038
AST (IU/L)	41(10–668)	39(10–138)	49(18–668)	0.067
T-Bil (mg/dl)	1.09(0.25–18.97)	1.05(0.25–7.41)	1.3(0.29–18.97)	0.236
Crea (mg/dl)	0.83(0.24–4.04)	0.87(0.42–4.04)	0.79(0.24–3.92)	0.657
INR	1.26(1.05–2.29)	1.24(1.06–2.01)	1.31(1.05–2.29)	0.120
PLT (X10^9^/L)	71(3-267)	74(3-267)	70(20–161)	0.787
High-risk EV				0.063
Yes	93(75%)	65(70.7%)	28(87.5%)	
No	31(25%)	27(29.3%)	4(12.5%)	
EV bleeding				0.006
Yes	17(13.7%)	8(8.7%)	9(28.1%)	
No	107(86.3%)	84(91.3%)	23(71.9%)	
NSBBs				0.212
Yes	52(41.9%)	42(45.7%)	10(31.2%)	
No	72(58.1%)	50(54.3%)	22(68.8%)	
Prophylactic program				0.106
Yes	101(81.5%)	78(84.8%)	23(71.9%)	
No	23(18.5%)	14(15.2%)	9(28.1%)	

HCC, hepatocellular carcinoma; ALT, alanine aminotransferase; AST, aspartate transaminase; T-Bil, total bilirubin; INR, international normalized ratio; PLT, platelet; EV, esophageal varice; NSBBs, non-selective beta blockers. Variables with Non-normal distribution median (minimum, maximum) and analyzed with the Mann–Whitney nonparametric test.

During the lockdown period, CTP class and the ALBI grade were better in those patients whose endoscopy was postponed. No EVBs were seen; there was a higher percentage of patients on NSBBs use in the postponed group than the EVL group (14/23, 60.8% vs. 10/32, 31.3%) (Table 2).

**Table 2:** Demographic data of patients according to priority of endoscopic management during the lockdown period

Patient Demographic	All(N = 55)	Prioritized (N = 32)	Postponed (N = 23)	*p* value
Age(years)	60(52–69)	69.5(64.2–74.7)	59(52–75)	0.360
Sex				0.391
Male	37(67.3%)	23(71.9%)	14(60.9%)	
Female	18(32.7%)	9(28.1%)	9(39.1%)	
HBsAg				0.783
Positive	18(32.7%)	10(31.3%)	8(34.8%)	
Negative	37(67.3%)	22(68.7%)	15(63.2%)	
Anti-HCV				0.435
Positive	7(12.7%)	3(10.3%)	4(17.4%)	
Negative	48(87.3%)	29(89.7%)	19(82.6%)	
Alcohol				0.742
Positive	12(21.8%)	8(25%)	4(17.4%)	
Negative	43(78.2%)	24(75%)	19(82.6%)	
MELD score > 10				0.391
Yes	37(67.3%)	23(71.9%)	14(60.9%)	
No	18(32.7%)	9(28.1%)	9(39.1%)	
CTP class				0.043
A	32(58.1%)	15(46.9%)	17(73.9%)	
B	17(30.9%)	11(34.4%)	6(26.1%)	
C	6(11.0%)	6(18.7%)	0	
ALBI grade				0.026
1	12(21.8%)	3(9.4%)	9(39.1%)	
2	38(69.1%)	25(78.1%)	13(56.5%)	
3	5(9.1%)	4(12.5%)	1(4.3%)	
PALBI grade				0.069
1	17(30.9%)	6(18.7%)	11(47.8%)	
2	20(36.4%)	14(43.8%)	6(26.1%)	
3	18(32.7%)	12(37.5%)	6(26.1%)	
HCC				0.562
Yes	10(18.2%)	5(15.6%)	5(21.7%)	
No	45(81.8%)	27(84.4%)	18(78.3%)	
Other malignancy				0.383
Yes	6(10.9%)	5(15.6%)	1(4.3%)	
No	49(89.1%)	27(84.4%)	22(95.7%)	
Biochemistry				
Albumin (g/dl)	3.5(3.1-4.0)	3.3(3.0- 3.7)	3.8(3.4–4.3)	0.004
ALT (IU/L)	30(19–44)	37(26–45)	26(17–33)	0.016
AST (IU/L)	42(28–70)	49(33–78)	34(25–46)	0.019
T-Bil (mg/dl)	1.29(0.81–1.64)	1.3(0.88–1.76)	1.26(0.73–1.62)	0.413
Crea (mg/dl)	0.82(0.67–0.99)	0.79(0.60–1.02)	0.85(0.72–0.99)	0.403
INR	1.28(1.18–1.43)	1.31(1.22–1.44)	1.24(1.13–1.43)	0.232
PLT (X10^9^/L)	78(56-109.1)	70(52–107)	94(63–139)	0.105
High-risk EV				< 0.001
Yes	28(50.9%)	28(87.5%)	0	
No	27(49.1%)	4(12.5%)	23(100%)	
EV bleeding				0.007
Yes	9(16.4%)	9(28.1%)	0	
No	46(83.6%)	23(71.9%)	23(100%)	
NSBBs				0.029
Yes	24(43.6%)	10(31.2%)	14(60.9%)	
No	31(56.4%)	22(68.8%)	9(39.1%)	
Prophylactic program				0.007
Yes	46(83.6%)	23(71.9%)	23(100%)	
No	9(16.4%)	9(28.1%)	0	

HCC, hepatocellular carcinoma; ALT, alanine aminotransferase; AST, aspartate transaminase; T-Bil, total bilirubin; INR, international normalized ratio; PLT, platelet; EV, esophageal varice; NSBBs, non-selective beta blockers. Variables with Non-normal distribution median (minimum, maximum) and analyzed with the Mann–Whitney nonparametric test.

### Factors associated with EVB

On univariable analysis of 124 patients undergoing EVL, ALBI grade > 1, PALBI grade > 1, CTP class C, high-risk EV, and EVL during lockdown were determinants of EVB. Use of NSBBs and entrance into the EVL prophylaxis program were protective factors for EVB In subgroup analysis of patients undergoing EVL during lockdown period, CTP class C and entrance to the EVL program were determinants of EVB in a univariable analysis (Table 3).

**Table 3:** The univariate analysis with variceal bleeding in patients undergoing EVL

			Total		Lock down period		
Variable	N		Hazard ratio (95% CI)	*p*	N	Hazard ratio (95% CI)	*P*
Age (y/o) > 65/≦65	50/74		1.811(0.647–5.038)	0.258	10/22	0.441(0.088–2.209)	0.319
Gender M/F	85/39		1.118(0.365–3.425)	0.845	23/9	0.347(0.067–1.801)	0.208
HBsAg Y/N	40/84		1.570(0.550–4.483)	0.400	10/22	2.267(0.453–11.349)	0.319
Anti-HCV Y/N	21/103		1.629(0.474–5.599)	0.439	3/29	1.312(0.104–16.556)	0.833
Alcoholism Y/N	26/98		0.461(0.098–2.159)	0.326	8/24	0.286(0.030–2.740)	0.277
HCC Y/N	35/89		1.467(0.497–4.330)	0.488	5/27	1.905(0.262–13.871)	0.525
Other malignancy Y/N	9/115		0.773(0.091–6.606)	0.814	5/27	0.594(0.057–6.175)	0.663
ALBI grade 2&3/1	94/30		5.949(0.755–46.896)	0.091	28/4	1.200(0.108–13.322)	0.882
PALBI grade 2&3/1	85/39		3.964(0.860-18.276)	0.077	6/26	0.737(0.110–4.955)	0.753
Platelet (ml^− 1^) ≦ 100 K />100 K	84/40		0.480(0.170–1.356)	0.166	23/9	0.347(0.067–1.801)	0.208
MELD > 10/≦10	73/51		1.810(0.596–5.499)	0.295	23/9	0.347(0.067–1.801)	0.208
CTP C/A&B	15/109		8.662(2.596–28.903)	< 0.001	6/26	8.400(1.186–59.493)	0.033
High-risk EVs Y/N	92/31		6.234(0.791–49.099)	0.082	28/4	1.200(0.108–13.322)	0.882
NSBBs Y/N	52/72		0.378(0.116–1.235)	0.107	10/22	0.194(0.021–1.829)	0.152
Prophylactic program Y/N	101/23		0.134(0.044–0.404)	< 0.001	22/10	0.016(0.001–0.222)	0.002
The lockdown period Y/N	92/32		4.109(1.426–11.838)	0.009			

HCC, hepatocellular carcinoma; NSBBs, non-selective beta blockers

## Discussion

This is the first study to describe the impact of mitigated endoscopy service on outcomes of patients with EV during lockdown. We found that EVB was more frequent during lockdown, but mainly in patients without previous EVL prophylaxis. For those with entrance to the EVL prophylaxis program, there was no higher risk of EVB, although some EVLs were postponed via triage policy during lockdown.

The triage strategy was to prioritize patients with high-risk varices for EVL and postpone endoscopy for those with low-risk or eradicated EV might decrease medical loading and reduce potential risk of COVID-19 transmission. Moreover, selected patients with high-risk EVs for EVL may prevent the potential risk of bleeding. It is worth noting that, after excluding referrals, EVB occurred in 6 of 116 (5.2%) patients during the normal period vs. 2 of 46 (4.3%) patients during lockdown, which indicates that the triage policy to postpone endoscopy for patients with low-risk varices was safe during the lockdown period under the COVID-19 pandemic.

Patients whose endoscopies postponed during lockdown had better liver function and a higher percentage of NSBBs prescriptions. This suggested EV eradication might be easier to achieve and maintain in patients under NSBBs or with better liver function.

Although triage policy finds it is safe to postpone endoscopy for patients with low-risk varices, it cannot be overemphasized that entrance into the prophylaxis program was equipotent for comprehensive triage. Increasing variceal bleeding during lockdown was mainly due to increased emergency visits of referred cases, who did not receive regular prophylaxis program at our hospital. During lockdown period, entrance into the prophylaxis program was the only protective factor for EVB. The result might be owing to the lower risk of variceal bleeding after sequential EVLs, as bleeding rarely occurred after variceal eradication [29]. Prophylaxis program of variceal bleeding in our hospital was based on AASLD guideline, which recommends following EGD 3 to 6 months after eradication and then every 6 to 12 months [16]. Our study demonstrated a successful application in real world and encourage a regular surveillance program.

There were several limitations in this study. First, small case numbers due to the level 3 epidemic warning period was only 10 weeks in Taiwan. Second, we found the implementation of the triage policy to postpone endoscopy was not associated with increased risk of bleeding, and entrance into the prophylaxis program was associated with decreased bleeding risk; however, a causal relationship cannot be established due to the lack of prospective comparison. Third, we did not know how many patients with high-risk varices, if without endoscopic detection, during lockdown had an impact on bleeding Forth, the number of variceal bleeding was limited in multi-variable analysis and the interpretation of the result should be cautious.

In summary, triage policy that postpones endoscopy for patients with low-risk varices was safe during lockdown. Entrance into the prophylaxis program did not only decrease the risk of EVB, but fostered triage measures that postponed endoscopy.

## Data Availability

The datasets used during the current study are available from the corresponding author upon reasonable request.
